# Social reproduction and health: contributions from Breilh and Lukács to social thinking in collective health

**DOI:** 10.1590/0034-7167-2024-0428

**Published:** 2025-07-11

**Authors:** Diego de Oliveira Souza

**Affiliations:** I Universidade Federal de Alagoas. Arapiraca, Alagoas, Brazil

**Keywords:** Changes, Social, Conditions, Social, Thinking, Public Health, Health., Condiciones Sociales, Estructura Social, Pensamiento, Salud Pública, Salud.

## Abstract

**Objective:**

To analyze the relationship between social reproduction and health based on the theories of Jaime Breilh and Georg Lukács.

**Methods:**

This is a theoretical-conceptual study grounded in the immanent analysis of works by these authors.

**Results:**

It was found that, for Breilh, social reproduction establishes the dimensional frameworks for the social determination of health: general, particular, and individual. The author also defines essential principles for transforming life: sustainability, sovereignty, solidarity, and integral security. Lukács, in turn, emphasizes the mediation between the reproduction of individuals and society, highlighting the primacy of labor, which produces use-value. Within this relationship, alienating mediations may emerge, leading to human degradation, including health impacts.

**Final considerations:**

Both perspectives offer elements for rethinking health practices. Despite their differences, they critique the structural inequalities inherent in the capitalist mode of production and their repercussions on health.

## RESUMEN

**Objetivo:**

Analizar la relación entre reproducción social y salud a partir de las teorías de Jaime Breilh y Georg Lukács.

**Métodos:**

Estudio teórico-conceptual fundamentado en el análisis inmanente de las obras de estos autores.

**Resultados:**

Para Breilh, la reproducción social establece los marcos dimensionales para la determinación social de la salud: general, particular e individual. Él también define principios para transformar la vida: sostenibilidad, soberanía, solidaridad y seguridad integral. Por su parte, Lukács enfatiza la mediación entre la reproducción de los individuos y de la sociedad, destacando la primacía del trabajo, que es productor de valor de uso. En esta relación, pueden interponerse mediaciones alienantes, determinando la degradación humana, incluidos los impactos en la salud.

**Consideraciones finales:**

Ambas perspectivas ofrecen elementos para repensar las prácticas de salud. A pesar de sus diferencias, critican la desigualdad estructural del modo de producción capitalista y sus repercusiones en la salud.

## INTRODUCTION

The relationship between social reproduction and health has occupied a significant place in the foundational debates of collective health since the 1970s and 1980s. Far from being merely a biological phenomenon, health is determined by social structure, with its dynamics shaped by the possibilities presented by social reproduction. This has been a theoretical category extensively studied within the Marxist framework, particularly in opposition to fragmented or mechanical explanations of forms of social interaction. Since Marx, a multidimensional understanding of social totality has emerged, profoundly linked to the sphere of economic production but not confined to it; on the contrary, it produces effects across all social complexes, including health^(1)^. Thus, this study seeks to relate social reproduction and health based on the theories of two prominent thinkers: Jaime Breilh and Georg Lukács.

Jaime Breilh is one of the pioneers of Latin American social medicine, later termed *collective health*. This field emerged from the exchange between health and social sciences, primarily engaging with Marxist perspectives. It opposes the old causal paradigm of public health/classical epidemiology, known as *monocausalism* or *multicausalism*. This paradigm relies on a probabilistic view of quantifiable causes, in which the social dimension appears external to the health-disease process, being treated as quantifiable and factorial.

Against this mode of thought, the Ecuadorian epidemiologist developed an approach that integrates collective health with social critique. His theory of the social determination of health emphasizes how living conditions, shaped by production relations, affect the health of populations. Breilh^(1,2)^ proposes a comprehensive view of the social dimension, understanding it as a totality that inherently includes health.

Like other Latin American social medicine intellectuals, Jaime Breilh contributed beyond the academic sphere, extending into the political arena, especially during the 1970s and 1980s. At the time, several Latin American countries were under military dictatorships, which were contested by leftist political movements. The theoretical-practical movement of introducing authors like Marx into the health debate represented the creation of one such space of resistance, with the potential to include activists from fields outside of health.

The Hungarian Georg Lukács, in turn, is a major figure in Marxism-considered by many as one of the greatest philosophers of the 20th century. While he did not study health directly, his reflections on social reproduction can contribute to the debate on the social determination of health. For Lukács^(3)^, social reproduction is a dialectical process that involves both the conservation and transformation of social structures, deeply interconnected with the relationship between the individual and society, and rooted in the process of labor.

Lukács was also active politically. He was one of the founders of the Hungarian Communist Party, participated in government in 1919, and was subsequently exiled after the defeat of the Hungarian Revolution. His alignment with communism following the 1917 Russian Revolution influenced his theoretical work, leading him away from Hegelian perspectives toward Marxism, with Lenin’s works playing a crucial role. This process culminated in Lukács’s self-critique of “History and Class Consciousness.” From then on, he sought to conceive a Marxist Ethics, which presupposed the elucidation of the ontology of social being. Although Lukács died before completing his intended analysis of Ethics, he left an important manuscript on the ontology of social being. This work includes a chapter on social reproduction, an indispensable discussion for critiquing positivism, vulgar Marxism, and irrationalism.

Given this brief contextualization of the two authors, it is worth investigating the convergences and divergences in their discussions of social reproduction, as well as the potential for advancing the interfaces with health.

## OBJECTIVE

To analyze the relationship between social reproduction and health based on the theories of Jaime Breilh and Georg Lukács.

## METHODS

This study employs a theoretical-conceptual methodology based on the immanent analysis of selected works by these authors to explore their contributions and identify points of convergence and divergence. Immanent analysis is a technique of immersion in the text, philosophically tied to the idea of immanence, which pertains to what is internal to the process and does not transcend it. This type of analysis allows for an understanding of the text’s internal structure, its central categories, and the connections among these categories, aiming to interpret the author’s thought as accurately as possible^(4)^.

Although Lukács’s work predates Breilh’s chronologically, the decision was made to first present the Ecuadorian’s arguments due to their direct connection to health. This approach aims to enhance the potential connections between health and Lukácsian categories.

The analysis of Breilh’s contributions draws from the book *Epidemiology: economy, politics, and health (Epidemiologia: economia, política e saúde)* ^(1)^, which contains much of the text from his master’s thesis, defended in 1976-a milestone in the debate on collective health. Additionally, an article^(5)^ and his recent book published by Oxford^(2)^, recognized in an editorial by *The Lancet* for its originality and critical perspective^(6)^, are also analyzed. For Lukács, the study uses *Toward an ontology of social being (Para uma ontologia do ser social)* ^(3)^, volume II, supported by expert commentators^(4,7,8)^, in which he conducts a systematic and exhaustive analysis of the category of “social reproduction.”

## RESULTS

### Social reproduction and health: contributions of Breilh

By proposing that health is socially determined, Breilh^(1)^ demonstrated how the contradictions between the development of productive forces and the social relations of production determine pauperization, social inequality, and, consequently, the degradation of human health. He aligns with the Marxian debate and demonstrates how economic elements, although central, are insufficient to explain the dynamics of social life in all its dimensions. At this point, he invokes the debates on totality and social reproduction, considering the influence of Marx and other contemporary critical authors.

Directly inspired by Echeverría^(9)^, Breilh^(1,2)^ defines *social reproduction* as the process through which life is produced and distributed, encompassing both the creation of goods and services and the ideological, cultural, and political conditions that sustain this process. Along these lines, Breilh^(2)^ understands the production of life as an articulation of production, distribution, and consumption, dynamized in three dialectically interdependent dimensions:

- General dimension: This dimension refers to the logic of capitalist accumulation and the hegemonic policies and cultures that sustain it and influence the other two dimensions.
- Particular dimension: This dimension addresses the reproduction of social classes, shaped by gender and ethnocultural relations. These relations may involve cooperation, exploitation, or domination. Health in this dimension is affected by the material and symbolic conditions in which distinct social groups develop, determining their ways of life.
- Individual dimension: This dimension includes individuals and families, with their lifestyles and daily lives. The class, gender, and ethnocultural conditions in which these individuals live directly and indirectly influence their health, affecting their bodies, organisms, phenotypes, genotypes, psychism, and forms of spirituality.

Indeed, the perspective of the social determination of health is crucial to understanding how health embodiments are generated across the different dimensions of social reproduction. Unlike determinism, which suggests a linear and direct causality, social determination advocates a dialectical and complex vision in which health results from interactions operating at different social and natural reality levels.

Beyond the three dimensions, Breilh^(2)^ highlights a contradiction in how social reproduction translates into human life since, under capitalism, there is an opposition between the individual and society. Drawing on contributions from Samaja^(10)^, another pioneer of collective health, Breilh^(2)^ underscores the dialectical nature of two major contradictory trends in society: on the one hand, the creative capacity within the particular domain, generating diversity and reflections in the generic sphere; on the other hand, a counter-movement by society as a whole, aiming to perpetuate an existence filled with inequalities that are continuously reinstated within social reproduction.

Considering these contradictory trends between individuals and society, the articulation of the dimensions of social reproduction leads Breilh^(2)^ to certain conclusions: health cannot be reduced to the simple sum of individual risk factors, rejecting the fragmenting tendency of positivism. This argument directly critiques what has been conventionally termed the *social determinants of health*, which at first glance appears close to what Breilh called the *social determination of health* but, in fact, represents a Cartesian imperative that separates social elements to quantify and associate them. Unlike determination, determinants do not consider the contradictory trends between individuals and society or the totality forged in articulating production, distribution, and consumption across multiple dimensions. Instead, they fragment and then attempt to mechanically reconnect these fragments.

Within the scope of totality, Breilh^(2)^ highlights the production of inequities as one of the references for the social determination of health. Therefore, he envisions a horizon of equity, encompassing the struggle against class, gender, and ethnic oppression. Health equity refers to economic complementarity and sufficiency, distributive justice, democratic empowerment, and non discrimination in all its forms. Conversely, inequity results from the concentration of power and resources in the hands of certain classes and social groups, leading to economic, social, political, cultural, and epistemic-scientific subjugation. These structural inequalities deeply influence individuals’ and collectives’ life and health opportunities, hindering good living^1^(2).

The construction of equity and good living, free from the aforementioned oppressions, traverses four principles, called the *4 S* of life:

1. Sustainability: Refers to the capacity to sustain and reproduce human and natural life in a lasting manner, ensuring the material and ecological conditions necessary for health.
2. Sovereignty: Implies autonomy in leading life and control over essential resources, enabling communities and nations to decide their own development and well-being.
3. Solidarity: Advocates for an equitable civilization and a protective logic for the common good; it promotes popular organization and psychological and spiritual fraternity, essential for social cohesion and collective health.
4. Security (integral biosecurity): Refers to creating protective spaces and processes that ensure human and ecological health, including both risk prevention and the promotion of healthy living conditions^2^.

Finally, for Breilh^(2)^, the approach to social reproduction as the driving axis of the social determination of health has profound implications for research and practice in the field of collective health. By redefining health as a complex socio-natural phenomenon, a more comprehensive vision is promoted that considers the inseparability of social and natural reality.

Moreover, this approach suggests the need for policies and practices that address the structural causes of health inequities. For the Ecuadorian author, such interventions should be guided by the “4 S,” pointing to the redistribution of resources, the promotion of social justice, and the creation of conditions that enable community autonomy and empowerment. Only in this way, according to Breilh^(2)^, will it be possible to advance toward a truly sustainable and equitable development model that promotes health comprehensively.

### Possibilities in the debate with Lukács

It is important to clarify that Lukácsian ontology has no connection with ancient or medieval ontology, where speculative practices about the essence of a world understood as objective existence, yet “immune” to transformation by subjects, predominated. Nor is it equivalent to other contemporary existentialist ontologies, which reduce the essence of processes to the subject’s subjectivity, existing solely in their own experience.

Beyond this, Lukács^(3)^ advocates for an organic articulation between objectivity and subjectivity, although objectivity is the predominant moment. In other words, the world exists objectively, independent of the degree of awareness subjects have of it. However, the production of these processes is entirely linked to the actions of subjects, through praxis, which opens the horizon for historical transformation depending on objective conditions and the awareness of these processes. Therefore, the essence of objects is not an immutable core but is instead understood as a radically historical essence of social being, produced by humans, although not at their whim.

Within this framework, the “social reproduction” category is pivotal for understanding the processes of social being. Lukács^(3)^ argues that social reproduction is a bipolar process. Its two inseparable poles are the reproduction of individuals (ontogenetic) and the reproduction of society as a whole (phylogenetic). This process reveals the interdependence between individual and societal development, which is always mediated.

It is worth noting that the articulation between individual and species reproduction is also present in the organic realm, as the ontogenetic reproduction of each individual organism contributes to the continuity of the species, though, in this case, without significant mediations or qualitative transformations. In the social sphere, this relationship becomes more complex. Lukács^(3)^ contends that the biological reproduction of human beings, while essential, is only a part of the larger process of social reproduction. Human ontogenetic reproduction is not limited to mere biological continuity; it also involves creating social and material conditions that sustain human life and enable its continuous development.

For Lukács^(3)^, the reproduction of inorganic being constantly transforms into another, and the reproduction of natural being (the biological sphere) always reestablishes the same (producing a new example of the species). Social reproduction, however, is different: it is always the creation and recreation of the new, that is, the simultaneous transformation of individuals and society.

Thus, social reproduction is fundamentally a process of mediation. Through their practical activities, humans interact with nature and other humans, creating and recreating the conditions for their existence. This process is dialectical, as it involves both the preservation and the transformation of social structures. Therefore, social reproduction is not merely a mechanical repetition of existing conditions but a dynamic process that includes the possibility of change and development^(7,8)^.

According to Andrade^(8)^, the economy is a crucial component of this dynamic, understood as the productive sphere through which humans create society via labor. The economic dimension bridges the reproduction of the human species and the individual, establishing the material conditions for human existence. The objective interaction within the social reproductive process fosters the development of productive forces and human capacities. This is because the outcome of the labor process exceeds use-value; it transforms the subject, creates new possibilities, and generates new needs, enriching the social being. However, this interaction can also lead to alienation and human degradation, depending on concrete social conditions. Specifically, this occurs when labor is based on exploitation and hinders individuals’ full connection with the human species.

The gulf created between individuals and the human species expresses the dehumanizing nature of a particular form of labor. Contradictorily, all forms of labor remain the foundational activity of social being and are therefore responsible for humanization (becoming human). This contradiction is realized when the production of use-values becomes subordinate to the production of exchange values, necessary for the process of capital valorization and, consequently, capital accumulation. Thus, commodities, human labor products, come to govern social relations.

Within this framework, one of the central concepts in Lukács’s analysis is the ontological primacy of use-value over exchange-value. He argues that use-value, which emerges from the satisfaction of human needs through labor (a permanent condition), has ontological precedence over exchange-value, which is specific to a historical phase. This primacy reflects the biological foundation of human reproduction, that is, the dimension in which labor products reveal their capacity to satisfy basic human needs-this being prior to and fundamental to all other forms of human activity. Consequently, the biological reproduction of human life forms the ontological basis for all other categories of social being, affirming their indissoluble interrelation^(8)^.

In this argument, the influence of Hegelian categories of universality, particularity, and singularity reappears, though reformulated through historical-dialectical materialism. This signifies that social reproduction represents the organic articulation between the singular dimension of ontogenetic reproduction and the universal dimension of phylogenetic reproduction. However, this articulation is not direct: it presupposes particularities that mediate the relationship.

As previously noted, the relationship between individual and society may manifest in a manner dominated by dehumanization. According to Lessa^(7)^, the particularity of alienation under capitalism occurs in two ways: first, human needs become increasingly irrelevant in social production; second, production becomes characterized by waste and destruction, evidencing the inhumanity of capital. This scenario leads to a social life marked by profound inequalities and uncertainties, where unemployment and precarity become central issues. Thus, the foundations and social dynamics degrading to health are forged, both individually and collectively.

Lessa^(7)^, following Lukács, perceives overcoming capitalism as a necessary condition for true human emancipation. The author argues that as long as the logic of capital orients production, humanity will remain alienated and subjected to an existence shaped by the commodification of social relations. Nevertheless, if reality is radically historical, humans can recreate social reality through their conscious actions and under specific objective conditions. The starting point for this process lies in that foundational dimension, the labor complex, which operates only through the essential mediations within social reproduction. Therefore, the critique of capitalist social reproduction is not merely an economic analysis but also a radical critique of the forms of life and human relations emerging from this system^(3,7,8)^.

Convergences in the understanding of social reproduction by Breilh and Lukács are evident. A shared point is the understanding of social reproduction in its various dimensions, through the dialectic of universal-particular-singular, even though Breilh demonstrates slight differences in vocabulary.

These two authors also diverge, particularly in the emphasis given to the analyzed processes and their strategies for addressing them. Breilh adapted his arguments over the years; initially (in the 1970s), he focused on economic inequality and the centrality of labor to decipher and confront issues within the framework of capitalism. Over time, this centrality has been complemented by other inequalities and oppressions, along with the “4 S” principles of social determination^(2,5)^.

For Lukács^(3)^, other inequalities and oppressions are essential but are understood from the perspective of their functions within the mode of production and their mediation of social reproduction. The centrality lies in labor, the foundational activity of social being, from which the fundamental contradiction of society originates. Social reproduction is understood through the articulation of its two poles (ontogenetic and phylogenetic), made possible by labor. It can be richer when more humanizing mediations exist or more degrading (including health degradation) when alienating mediations intervene between individuals and the human species.

Thus, Lukács’s theory can contribute to the debate on the relationship between social reproduction and health, particularly because it demonstrates biological reproduction as a prerequisite for social reproduction. This condition translates into the primacy of use-value, a Marxian category explaining the character of labor products in meeting human needs. Both reproductions (biological and social) are undermined under capitalism, as use-value is subordinated to exchange-value (and human needs, consequently, to market needs). Therefore, social reproduction becomes centered on pauperism on one hand and capital accumulation on the other.

Labor cannot be concealed by any social mediations; instead, it must be understood as the heuristic key to comprehending them. This in no way diminishes the importance of these mediations, as Lukács^(3)^ attributes to them the ability to enrich social reproduction. Furthermore, Lukács’s theory opens the possibility of understanding and transforming these mediations into genuinely humanizing elements capable of forging authentic connections between individuals and the human species, a strategy that necessarily involves class struggle and the transcendence of the capitalist system.

## FINAL CONSIDERATIONS

The analysis of Jaime Breilh and Georg Lukács’s theories on social reproduction reveals the depth and complexity of this topic. Breilh develops a multifaceted approach, integrating social reproduction’s general, particular, and individual dimensions. He identifies the “4 S” as fundamental pillars for a healthy life, advocating for policies that address the structural causes of health inequities. On the other hand, Lukács offers a dialectical perspective on social reproduction, focusing on the mediation between individual and societal development. He argues that social reproduction is not merely a process of repetition but also a dynamic field that can lead to either alienation or emancipation, depending on concrete social conditions and humanity’s awareness of its historical paths.

Breilh successfully integrates perspectives broadly embedded in the critical field, including Marxism and Andean philosophy. Meanwhile, Lukács situates himself within a more orthodox (in the sense of precise interpretation) framework of Marxism, contributing to critiques of interpretations that fail to grasp the essence of social reproduction. Both perspectives offer insights for rethinking health (and health practices). Despite their differences, they critique the structural inequalities of the capitalist mode of production and its repercussions on health.

## Footnotes

1
*Good living* is a concept rooted in the philosophy of Indigenous Andean peoples. Good living transcends the idea of individual well-being to encompass collective harmony with nature and among human beings, emphasizing the importance of a dignified and balanced life in all its dimensions. It entails integration with nature, equity and justice, autonomy and community participation, cultural and identity sovereignty, and economic relations grounded in solidarity^(1)^. This concept underpins what Breilh referred to as the *4 S*.

2 The concept of *good living* envisions a society structured around the “4 S” principles, marking one of its distinctions from Marx’s social theory. From a Marxist perspective, the primary critique lies in the fact that the “4 S” principles overshadow the mode of production (as articulated through social reproduction) as the foundational basis of society. This condition ultimately displaces the ontologically foundational role of labor in relation to society and, consequently, may hinder the understanding of the need to overcome the contradiction between the development of the means of production and social relations of production. Therefore, the issue does not lie in the development of the means of production itself but rather in its occurrence within an antagonistic relationship between capital and labor.
