# Supplementary material for: Social reproduction and health: contributions from Breilh and Lukács to social thinking in collective health
Source: Rev Bras Enferm. 2025 Jul 11;78(3):e20240428. doi: 10.1590/0034-7167-2024-0428 (PMC12252920; doi:10.1590/0034-7167-2024-0428)
Supplement: Translation

# Translation: Reprodução social e saúde: contribuições de Breilh e Lukács ao pensamento social em saúde coletiva

**DOI:** 10.1590/0034-7167-2024-0428pt

**Authors:** Diego de Oliveira Souza

**Affiliations:** I Universidade Federal de Alagoas. Arapiraca, Alagoas, Brasil

**Keywords:** Condições Sociais, Estrutura Social, Pensamento, Saúde Coletiva, Saúde

## Abstract

**Objetivo:**

Analisar a relação entre reprodução social e saúde apoiando-se nas teorias de Jaime Breilh e Georg Lukács.

**Métodos:**

Trata-se de um estudo teórico-conceitual baseado na análise imanente de obras desses autores.

**Resultados:**

Constatou-se que, para Breilh, a reprodução social estabelece os marcos dimensionais para a determinação social da saúde: geral, particular e individual. O autor ainda define os princípios essenciais para a transformação da vida: sustentabilidade, soberania, solidariedade e segurança integral. Por sua vez, Lukács enfatiza a mediação entre a reprodução dos indivíduos e da sociedade, destacando a primazia do trabalho, que é produtor de valor de uso. Nessa relação, podem ser interpostas mediações alienantes, determinando a degradação humana, incluindo impactos na saúde.

**Considerações finais:**

Ambas as perspectivas oferecem elementos para repensar as práticas de saúde. Apesar de suas diferenças, criticam a desigualdade estrutural do modo de produção capitalista e suas repercussões na saúde.

## INTRODUÇÃO

A relação entre reprodução social e saúde tem ocupado espaço relevante nos debates inaugurais da saúde coletiva desde os anos 1970 e 1980. Longe de ser apenas um fenômeno biológico, a saúde é determinada pela estrutura social, com uma dinâmica que se dá nas possibilidades postas pela reprodução social. Esta tem sido uma categoria teórica largamente estudada no campo marxista, opondo-se especialmente às explicações fragmentárias ou mecânicas sobre as formas de interação social. Desde Marx, engendra-se um entendimento de totalidade social multidimensional e profundamente articulada à esfera da produção econômica, mas que não se esgota nela; ao contrário, produz efeitos em todos os complexos sociais, incluindo a saúde^(1)^. Diante disso, este estudo busca relacionar reprodução social e saúde apoiando-se nas teorias de dois importantes pensadores: Jaime Breilh e Georg Lukács.

Jaime Breilh é um dos pioneiros da medicina social latino-americana, que depois veio a ser chamada “saúde coletiva”. Trata-se de um campo que nasce do intercâmbio entre ciências da saúde e ciências sociais, dialogando sobretudo com as perspectivas marxistas. Esse campo se opõe ao velho paradigma causal da saúde pública/epidemiologia clássica, conhecido como “monocausalismo” ou “multicausalismo”. Tal paradigma baseia-se na visão probabilística de causas quantificáveis, na qual a dimensão social aparece como algo externo ao processo saúde-doença, sendo considerada passível de quantificação e fatorialização.

Contra essa forma de pensar, o epidemiologista equatoriano desenvolveu uma abordagem que integra a saúde coletiva com a crítica social. Sua teoria da determinação social da saúde enfatiza como as condições de vida, moldadas pelas relações de produção, afetam a saúde das populações. Breilh^(1,2)^ propõe uma visão abrangente da dimensão social, entendendo-a como uma totalidade, logo inclui a saúde.

Assim como outros intelectuais da medicina social latino-americana, Jaime Breilh trouxe contribuições para além da esfera acadêmica, alcançando a esfera política, sobretudo entre os anos de 1970 e 1980. À época, vários países latino-americanos passavam por ditaduras militares, que eram contestadas por movimentos políticos à esquerda. O movimento teórico-prático de trazer autores como Marx para o debate da saúde representou a construção de um desses espaços de resistência, com potencial de abranger militantes de outros campos que não da saúde.

Já o húngaro Georg Lukács é uma referência do marxismo em geral - para muitos, um dos maiores filósofos do século XX. Ele não estudou a saúde diretamente, mas as suas reflexões sobre a reprodução social podem contribuir para o debate da determinação social da saúde. Para Lukács^(3)^, a reprodução social é um processo dialético que envolve tanto a conservação quanto a transformação das estruturas sociais, em uma profunda articulação entre indivíduo e sociedade, fundada no processo de trabalho.

Lukács também teve atuação política. Foi um dos fundadores do Partido Comunista Húngaro, participou do governo em 1919 e, inclusive, foi exilado após a derrota da Revolução Húngara. Sua aproximação com o comunismo após a Revolução Russa de 1917 repercutiu em sua produção teórica, quando se afasta da perspectiva de Hegel e se direciona ao marxismo, com as obras de Lenin desempenhando um papel relevante. Esse processo desemboca na autocrítica que o húngaro faz de “História e Consciência de Classe”. Desde então, veio a busca por pensar uma Ética marxista, que pressupunha a elucidação da ontologia do ser social. Lukács veio a morrer antes de concluir a pretendida análise sobre a Ética, mas deixou um importante manuscrito sobre a ontologia do ser social. Esse trabalho inclui um capítulo sobre reprodução social, discussão indispensável à crítica ao positivismo, ao marxismo vulgar e ao irracionalismo.

Considerando essa breve contextualização sobre os dois autores, convém investigar quais as convergências e divergências no debate sobre a reprodução social realizado pelos dois autores e as possibilidades de avanço nas interfaces com a saúde.

## OBJETIVO

Analisar a relação entre reprodução social e saúde apoiando-se nas teorias de Jaime Breilh e Georg Lukács.

## MÉTODOS

Utiliza-se uma metodologia teórico-conceitual, baseada na análise imanente de algumas obras desses autores, para explorar suas contribuições e identificar pontos de convergência e divergência. A análise imanente é uma técnica de imersão no texto filosoficamente ligada à ideia de imanência, ou seja, àquilo que é interno ao processo e não o transcende. Esse tipo de análise permite entender a estrutura interna do texto, as suas categorias centrais, os nexos na articulação entre as categorias, a fim de decifrar o pensamento do autor da forma mais aproximada possível^(4)^.

Embora cronologicamente a obra de Lukács anteceda a de Breilh, optou-se por expor primeiro os argumentos do equatoriano, por apresentar uma relação direta com a saúde. Didaticamente, esse caminho potencializará as conexões possíveis entre a saúde e as categorias lukacsianas.

De Breilh, utiliza-se o livro “Epidemiologia: economia, política e saúde”^(1)^, que traz boa parte do texto de sua dissertação de mestrado defendida em 1976, marco para o debate da Saúde Coletiva. Além disso, analisa-se um artigo^(5)^ e o seu recente livro publicado em Oxford^(2)^, reconhecido em editorial da The Lancet por seu caráter original e crítico^(6)^. De Lukács, faz-se uso da obra “Para uma ontologia do ser social”^(3)^, volume II, com suporte de comentadores especialistas^(4,7,8)^, na qual ele faz uma análise sistemática e exaustiva da categoria “reprodução social”.

## RESULTADOS

### Reprodução social e saúde: contribuições de Breilh

Ao postular a ideia de que a saúde é determinada socialmente, Breilh^(1)^ demonstrou como as contradições entre o desenvolvimento das forças produtivas e as relações sociais de produção determinam a pauperização, desigualdade social e, por conseguinte, a degradação da saúde humana. Ele se aproxima do debate marxiano e demonstra como os elementos econômicos, embora nevrálgicos, não eram suficientes para explicar a dinâmica da vida social em todas as suas dimensões. É nesse ponto que ele evoca o debate de totalidade e reprodução social, considerando a influência de Marx e de outros autores do campo crítico contemporâneo.

Diretamente inspirado por Echeverría^(9)^, Breilh^(1,2)^ define a “reprodução social” como o processo no qual se dá a produção e a distribuição da vida, abrangendo tanto a criação das mercadorias e serviços quanto as condições ideológicas, culturais e políticas que sustentam esse processo. Nessa linha, Breilh^(2)^ entende a produção da vida como uma articulação entre produção, distribuição e consumo, dinamizadas em três dimensões dialeticamente interdependentes:

- Dimensão geral: Esta dimensão refere-se à lógica de acumulação capitalista e às políticas e culturas hegemônicas que a sustentam e influenciam as outras duas dimensões.
- Dimensão particular: Aqui se aborda a reprodução das classes sociais, atravessadas por relações de gênero e etnoculturais. Essas relações podem ser de cooperação ou de exploração e dominação. A saúde nessa dimensão é afetada pelas condições materiais e simbólicas em que se desenvolvem os distintos grupos sociais, o que determina os modos de vida.
- Dimensão individual: Esta dimensão inclui os indivíduos e famílias, com seus estilos de vida e cotidiano. As condições de classe, gênero e etnoculturais em que vivem esses indivíduos influenciam sua saúde de maneira direta e indireta, afetando seu corpo, organismo, fenótipo, genótipo, psiquismo e formas de espiritualidade.

Com efeito, a perspectiva de determinação social da saúde é crucial para entender como são geradas as formas de corporificação da saúde nas diferentes dimensões da reprodução social. Diferente do determinismo, que sugere uma causalidade linear e direta, a determinação social advoga uma visão dialética e complexa, na qual a saúde é vista como o resultado de interações que operam em diferentes níveis da realidade social e natural.

Além das três dimensões, Breilh^(2)^ sublinha a existência de uma contradição na forma pela qual a reprodução social traduz-se em vida humana, porquanto no capitalismo há uma oposição entre indivíduo e sociedade. Com base nas contribuições de Samaja^(10)^, outro pioneiro da Saúde Coletiva, Breilh^(2)^ destaca a natureza dialética das duas grandes tendências contraditórias da sociedade: por um lado, a capacidade criativa existente no domínio particular, que gera diversidade e reflexos na esfera genérica; e, por outro lado, um movimento contrário por parte da sociedade em seu conjunto, no sentido de perpetuar uma existência repleta de desigualdades, recolocadas continuamente na reprodução social.

Considerando também as tendências contraditórias entre indivíduos e sociedade, a articulação das dimensões da reprodução social conduz Breilh^(2)^ a algumas constatações: a saúde não pode ser reduzida à simples soma de fatores de risco individuais, rechaçando a tendência fragmentante do positivismo. Esse argumento é uma crítica direta ao que se convencionou chamar de “determinantes sociais da saúde”, que, à primeira vista, parece se aproximar do que Breilh denominou “determinação social da saúde”, mas, na verdade, representa o imperativo cartesiano que separa os elementos sociais, para poder contabilizá-los e associá-los. Diferentemente da determinação, os determinantes não consideram as tendências contraditórias entre indivíduo e sociedade nem a totalidade forjada na articulação entre produção, distribuição e consumo, com as suas múltiplas dimensões. Em vez disso, eles fragmentam para depois tentar reconectar, mecanicamente, os fragmentos.

No bojo da totalidade, Breilh^(2)^ destaca a produção das iniquidades como um dos referenciais da determinação social da saúde. Portanto, vislumbra um horizonte de equidade, perpassando a luta contra as opressões de classe, gênero e etnia. A equidade em saúde refere-se à complementaridade e suficiência econômica, justiça distributiva, empoderamento democrático e não discriminação em todas as suas formas. Contrariamente, a iniquidade é o resultado da concentração de poder e recursos nas mãos de certas classes e grupos sociais, o que leva às relações de submissão econômica, social, política, cultural e epistêmico-científica. Essas desigualdades estruturais influenciam profundamente as oportunidades de vida e saúde dos indivíduos e coletivos, obstando o bem-viver^1 (2)^.

A construção da equidade e do bem-viver, livre das já citadas opressões, perpassam quatro princípios, denominados os “4 S” da vida:

1. Sustentabilidade - Refere-se à capacidade de manter e reproduzir a vida humana e natural de maneira duradoura, assegurando as condições materiais e ecológicas necessárias para a saúde.
2. Soberania - Implica a autonomia na condução da vida e o controle sobre os recursos indispensáveis, permitindo às comunidades e nações decidir sobre seu próprio desenvolvimento e bem-estar.
3. Solidariedade - Este princípio advoga uma civilização equitativa e uma lógica protetora para o bem comum; promove a organização popular e a fraternidade psicológica e espiritual, essenciais para a coesão social e saúde coletiva.
4. Segurança integral - Refere-se à criação de espaços e processos protetores que assegurem a saúde humana e ecológica, incluindo tanto a prevenção de riscos quanto a promoção de condições de vida saudáveis^2^.

Por fim, para Breilh^(2)^, a abordagem da reprodução social como eixo movente da determinação social da saúde tem profundas implicações para a pesquisa e prática no campo da saúde coletiva. Ao redefinir a saúde como um fenômeno socionatural complexo, promove-se uma visão mais integral que considera a indissociabilidade entre a realidade social e a natural.

Além disso, essa abordagem sugere a necessidade de políticas e práticas que tratem das causas estruturais da iniquidade em saúde. Para o equatoriano, esse tipo de intervenção deve pautar-se nos “4 S”, o que aponta para uma redistribuição de recursos, promoção da justiça social e criação de condições que permitam a autonomia e o empoderamento das comunidades. Só assim, segundo Breilh^(2)^, será possível avançar para um modelo de desenvolvimento realmente sustentável e equitativo, que promova a saúde de maneira integral.

### Possibilidades no debate com Lukács

É preciso esclarecer que a ontologia lukacsiana nada tem a ver com a ontologia antiga ou medieval, nas quais imperava a prática especulativa sobre a essência de um mundo que era entendido como existência objetiva, mas “imune” à transformação operada pelos sujeitos. Tampouco se equivale a outras ontologias contemporâneas, de cunho existencialista, as quais reduzem a essência dos processos à subjetividade do sujeito, que só existe na experiência em si.

Para além disso, Lukács^(3)^ defende uma orgânica articulação entre objetividade e subjetividade, embora o momento predominante seja o da objetividade. Em outras palavras, o mundo existe objetivamente, independentemente do grau de consciência que os sujeitos tenham sobre isso. Porém, a produção desses processos está totalmente consignada à ação dos sujeitos, na práxis, o que abre um horizonte de transformação da história a depender das condições objetivas e da tomada de consciência sobre os processos. Portanto, não se trata de falar de uma essência dos objetos como um núcleo imutável, mas de entender que a essência do ser social é radicalmente histórica, produzida pelos seres humanos, embora não ao seu bel prazer.

Nessa concatenação, a categoria “reprodução social” é decisiva para entender a processualidade do ser social. Lukács^(3)^ afirma que a reprodução social é um processo bipolar. Os dois polos inseparáveis são a reprodução dos indivíduos (ontogenética) e a reprodução da sociedade como um todo (filogenética). Esse processo revela a interdependência entre o desenvolvimento individual e o desenvolvimento societal, sempre mediatizado.

Convém fazer a ressalva de que a articulação entre reprodução do indivíduo e reprodução da espécie também está presente no reino orgânico, pois a reprodução ontogenética de cada organismo individual contribui para a continuidade da espécie, mas, nesse caso, sem grandes mediações ou transformações qualitativas entre tais processos. Na esfera social, essa relação torna-se mais complexa. Lukács^(3)^ argumenta que a reprodução biológica dos seres humanos, embora essencial, é apenas uma parte do processo maior de reprodução social. A reprodução ontogenética humana não se limita à mera continuação biológica; ela envolve também a criação de condições sociais e materiais que sustentam a vida humana e permitem seu desenvolvimento contínuo.

Constata-se que, para Lukács^(3)^, a reprodução do ser inorgânico é um constante transformar se em outro, e a reprodução do ser natural (a esfera biológica) é sempre uma reposição do mesmo (dar origem a um novo exemplar da espécie). Porém, a reprodução social é outra coisa: é sempre a criação e recriação do novo, ou seja, a transformação concomitante do indivíduo e da sociedade.

Assim, a reprodução social é essencialmente um processo de mediação. Os seres humanos, mediante suas atividades práticas, interagem com a natureza e com os outros seres humanos, criando e recriando as condições de sua existência. Esse processo é dialético, pois envolve tanto a conservação quanto a transformação das estruturas sociais. Portanto, a reprodução social não é apenas uma repetição mecânica das condições existentes, mas também um processo dinâmico que inclui a possibilidade de mudança e desenvolvimento^(7,8)^.

Segundo Andrade^(8)^, a economia é um componente crucial dessa dinâmica, entendida enquanto esfera produtiva a partir da qual os seres humanos produzem a sociedade por meio do trabalho. A dimensão econômica articula a reprodução do gênero humano e a reprodução do indivíduo singular, estabelecendo as condições materiais para a existência humana. Com efeito, a interação objetiva dentro do processo reprodutivo social promove o desenvolvimento das forças produtivas e das capacidades humanas. Isso porque o resultado do processo de trabalho ultrapassa o valor de uso, já que transforma o sujeito, cria novas possibilidades e gera novas necessidades, enriquecendo o ser social. No entanto, essa interação também pode levar à alienação e à degradação do ser humano, dependendo das condições sociais concretas: a rigor, isso ocorre quando o trabalho baseia-se na exploração e obsta a plena conexão dos indivíduos com o gênero humano.

O abismo criado entre os indivíduos e o gênero humano expressa o caráter desumanizador de uma forma particular de trabalho, embora, contraditoriamente, qualquer forma de trabalho mantenha se como a atividade fundante do ser social, logo, responsável pela humanização (tornar-se humano). Essa contradição efetiva-se quando a produção de valores de uso fica subordinada à produção de valores de troca, necessários para o processo de valorização de capital e, consequentemente, acumulação de capital. Assim, as mercadorias, produto da mão humana, passam a reger as relações sociais.

Considerando essa abordagem, um dos conceitos centrais na análise de Lukács é a prioridade ontológica do valor de uso sobre o valor de troca. Ele argumenta que o valor de uso, o qual emerge da satisfação das necessidades humanas por meio do trabalho (condição permanente), tem primazia ontológica sobre o valor de troca, algo específico de uma fase histórica. Essa primazia reflete a base biológica da reprodução humana, ou seja, a dimensão na qual os produtos de trabalho revelam sua capacidade de satisfazer as necessidades humanas básicas - o que é anterior e fundamental para todas as outras formas de atividade humana. Portanto, a reprodução biológica da vida humana forma a base ontológica de todas as outras categorias do ser social, ratificando o entendimento de indissociabilidade entre elas^(8)^.

Nessa argumentação, ressurge a influência das categorias hegelianas da universalidade, particularidade e singularidade, embora reformuladas com base no materialismo histórico-dialético. Isso significa que a reprodução social representa a orgânica articulação entre a dimensão singular da reprodução ontogenética com a dimensão universal da reprodução filogenética. Porém, essa articulação não é direta: ela pressupõe as particularidades que fazem a mediação da relação.

Como já destacado, tal relação entre indivíduo e sociedade pode consubstanciar-se de forma que predomine a desumanização. A particularidade da alienação no capitalismo, segundo Lessa^(7)^, ocorre em dois sentidos: primeiro, as necessidades humanas tornam-se cada vez menos relevantes na produção social; segundo, a produção passa a ser caracterizada por desperdício e destruição, evidenciando a desumanidade do capital. Esse quadro leva a uma vida social marcada por profundas desigualdades e incertezas, em que o desemprego e a precariedade tornam-se questões centrais. Dessa maneira, forjam-se as bases e as dinâmicas sociais degradantes para a saúde, seja individualmente, seja coletivamente.

Percebe-se que Lessa^(7)^, após Lukács, vê a superação do capitalismo como uma condição necessária para a verdadeira emancipação humana. O autor argumenta que, enquanto a produção for orientada pela lógica do capital, a humanidade permanecerá alienada e subordinada a uma existência marcada pela mercantilização das relações sociais. Contudo, se a realidade é radicalmente histórica, os seres humanos, por sua ação consciente e em determinadas condições objetivas, podem recriar a realidade social. O ponto de partida para esse processo está naquela dimensão fundante, o complexo do trabalho, mas que só opera devido às mediações imprescindíveis para isso na reprodução social. A crítica à reprodução social capitalista, portanto, não é apenas uma análise econômica, mas também uma crítica radical às formas de vida e às relações humanas que emergem desse sistema^(3,7,8)^.

Constatam-se convergências no entendimento de reprodução social de Breilh e Lukács. Um ponto comum é entender a reprodução social nas suas várias dimensões, por meio da dialética do universal-particular-singular, ainda que Breilh apresente uma pequena diferença no uso do vocabulário.

Esses dois autores também divergem, sobretudo nas ênfases dadas aos processos analisados e na estratégia de enfrentamento. Breilh foi adequando a sua argumentação ao longo dos anos, pois, inicialmente (década de 1970), ele enfatizava o debate sobre a desigualdade econômica e centralidade do trabalho para decifrar e enfrentar a problemática no marco do capitalismo. Ao longo dos anos, essa centralidade foi dividindo espaço com outras desigualdades e opressões, assim como também os pilares (princípios da vida) da determinação social passaram a ser os “4 S”^(2,5)^.

Para Lukács^(3)^, as demais desigualdades e opressões são essenciais. Porém, ele as entende sob a perspectiva das funções que exercem perante o modo de produção e, assim, de como intermedeiam a reprodução social. A centralidade está no trabalho, atividade fundante do ser social, da qual se origina a contradição fundamental da sociedade. A reprodução social é entendida pela articulação de seus dois polos (ontogenético e filogenético), tornada possível pelo trabalho. Ela pode ser tanto mais rica quanto mais mediações humanizadoras existam; e tanto mais degradante (incluindo a degradação da saúde) quanto mais mediações alienantes se interponham entre indivíduo e gênero humano.

Assim, a teoria de Lukács pode contribuir com o debate sobre a relação entre reprodução social e saúde, sobretudo porque demonstra a reprodução biológica como pressuposto da reprodução social. Essa condição se traduz na primazia do valor de uso, categoria marxiana que explica o caráter de os produtos do trabalho satisfazerem as necessidades humanas. Ambas as reproduções (biológica e social) são prejudicadas no capitalismo, já que o valor de uso fica subordinado ao valor de troca (logo, as necessidades humanas ficam subordinadas às necessidades do mercado). Assim, a reprodução social passa a ser constituída ao redor do pauperismo, de um lado; e da acumulação de capital, do outro.

O trabalho não pode ser uma categoria ocultada por quaisquer mediações sociais, e sim deve ser compreendido como a chave heurística para entendê-las. De modo algum, tal fato diminui a importância dessas mediações, visto que Lukács^(3)^ atribui a elas a capacidade de enriquecer a reprodução social. Para além disso, a teoria lukacsiana abre a possibilidade de entender e transformar essas mediações em elementos realmente humanizadores, capazes de tecer conexões autênticas entre indivíduos e gênero humano, o que, estrategicamente, perpassa a luta de classe e a superação do sistema do capital.

## CONSIDERAÇÕES FINAIS

A análise das teorias de Jaime Breilh e Georg Lukács acerca da reprodução social revela a profundidade e complexidade desse tema. Breilh desenvolve uma abordagem multifacetada, integrando dimensões gerais, particulares e individuais da reprodução social. Ele identifica os “4 S” como pilares fundamentais para uma vida saudável, evocando políticas que abordem as causas estruturais da iniquidade em saúde. Por outro lado, Lukács oferece uma visão dialética da reprodução social, focando na mediação entre o desenvolvimento individual e societal. Ele argumenta que a reprodução social não é apenas um processo de repetição, mas também um campo dinâmico que pode levar tanto à alienação quanto à emancipação, dependendo das condições sociais concretas e da tomada de consciência da humanidade sobre os caminhos de sua história.

Breilh consegue integrar perspectivas pluralmente inseridas no campo crítico, incluindo o marxismo e a filosofia andina. Já Lukács se situa no âmbito mais ortodoxo (no sentido de interpretação precisa) do marxismo, contribuindo para a crítica aos marxismos que não alcançaram o âmago do que seja a reprodução social. Ambas as perspectivas oferecem elementos para repensar a saúde (e as práticas de saúde). Apesar de suas diferenças, criticam a desigualdade estrutural do modo de produção capitalista e suas repercussões na saúde.

## Footnotes

1 O bem-viver é um conceito baseado na filosofia de povos originários andinos. O bem-viver transcende a ideia de bem-estar individual para incluir uma harmonia coletiva com a natureza e entre os seres humanos, ressaltando a importância de uma vida digna e equilibrada em todas as suas dimensões. Pressupõe integração com a natureza, equidade e justiça, autonomia e participação comunitária, soberania cultural e identitária e relações econômicas baseadas na solidariedade^(1)^. Esse conceito é a base para o que Breilh denominou de “4 S”.

2 A ideia de bem-viver projeta uma sociedade estruturada a partir dos “4 S”, o que demarca uma de suas diferenças com a teoria social de Marx. Do ponto de vista marxista, a principal crítica consiste no fato de que os “4 S” se sobrepõem ao modo de produção (articulado à reprodução social) como base estruturante da sociedade. Essa condição acaba por deslocar o papel ontologicamente fundante do trabalho para com a sociedade e, consequentemente, pode dificultar o entendimento da necessidade de superação da contradição entre o desenvolvimento dos meios de produção e as relações sociais de produção. Portanto, o problema não reside no desenvolvimento dos meios de produção em si, mas na sua ocorrência dentro de uma relação antagônica entre capital e trabalho.
